# A Comparison of the Incidences of Venous Thromboembolism after Total Hip Arthroplasty between the Direct Anterior Approach and the Direct Lateral Approach, Especially in the Early Period after Introduction of the Direct Anterior Approach

**DOI:** 10.1155/2020/4649207

**Published:** 2020-06-03

**Authors:** Tetsuya Kawano, Hiroaki Kijima, Shin Yamada, Natsuo Konishi, Hitoshi Kubota, Hiroshi Tazawa, Takayuki Tani, Norio Suzuki, Keiji Kamo, Yoshihiko Okudera, Masashi Fujii, Ken Sasaki, Yosuke Iwamoto, Itsuki Nagahata, Takanori Miura, Naohisa Miyakoshi, Yoichi Shimada

**Affiliations:** ^1^Department of Orthopedic Surgery, Akita University Graduate School of Medicine, Akita, Japan; ^2^Akita Hip Research Group (AHRG), Akita, Japan

## Abstract

**Objective:**

To compare the incidence of venous thromboembolism (VTE) after total hip arthroplasty (THA) using the direct anterior approach (DAA) with that using the direct lateral approach (DLA). In addition, patient background characteristics and the incidence of VTE were compared between the first half and the latter half of the period after introducing DAA and against DLA.

**Method:**

This was a retrospective, multicenter study involving 109 patients (116 hips) who had undergone primary unilateral THA. Thirty-six hips underwent THA using DAA and 80 hips underwent THA using DLA. Patient information including sex, age, and preoperative diagnosis was collected. The incidence of VTE was compared between DAA and DLA. Moreover, the patients who underwent THA using DAA were divided into 2 groups (first half and latter half groups), and sex, age, body mass index (BMI), and surgical time were compared between the 2 groups. Moreover, the incidence of VTE was compared among the 3 groups (first half of DAA, latter half of DAA, and DLA).

**Results:**

The incidence of VTE in the DAA group was significantly higher than that in the DLA group (*p*=0.014). The incidence of VTE in the first half group was significantly higher than in the latter half group and the DLA group (*p*=0.035 and *p*=0.001, respectively), and there was no difference in the incidence of VTE between the latter half group and the DLA group (*p*=0.923). Surgical time was significantly longer in the first half group than in the latter half group (*p*=0.046).

**Conclusions:**

In the first half of the period after introducing the DAA, more VTEs occurred than in the DLA. It may be important to shorten the surgical time in the early stage of introducing the DAA, and aggressive anticoagulation therapy may be required until the surgeon becomes familiar with the procedure.

## 1. Introduction

Venous thromboembolism (VTE) is a generic term for pulmonary embolism (PE) and deep venous thrombosis (DVT), and it is a serious complication that can occur after total hip arthroplasty (THA). Because the mortality rate of symptomatic PE is high, the prevention of VTE is very important. This includes anticoagulation therapy and physical prevention; however, when anticoagulation therapy is given to the patients with a risk of hemorrhage, aged patients, and those with impaired renal function, attention must be paid to the occurrence of hemorrhagic complications [1]. On the other hand, physical methods of prevention, such as early postoperative mobilization, have a low cost and a low risk of complications. Therefore, the American Academy of Orthopaedic Surgeons (AAOS) guidelines recommended physical prevention for preventing VTE [2].

There are several approaches to THA. The direct anterior approach (DAA) uses a natural intramuscular and intranerve interval [3]. This approach can lead to more rapid recovery after surgery because it is less invasive. From August 2015, we changed the standard approach for THA from a direct lateral approach (DLA), including the Dall and Hardinge approach, to DAA. However, we had many cases of VTE immediately after introduction of DAA, in contrary to our expectations. Thus, the aim of this study was to compare the incidence of VTE after THA using DAA with that using DLA. In addition, the incidence of VTE in two time periods after introducing the DAA was investigated.

## 2. Materials and Methods

This was a retrospective, multicenter study. Patients were recruited from January 2014 to November 2017. Of these cases, patients who underwent bilateral THAs on the same day, underwent revision THA, underwent THA after osteotomy, or underwent THA due to fracture were excluded. A total of 109 patients (116 hips) who had undergone primary unilateral THA were included. One surgeon performed THA using DAA (36 hips), and 3 experienced surgeons performed THA using DLA (Dall approach, 62 hips; Hardinge approach, 18 hips). THAs using DAA and DLA were performed with the patient placed in the supine position and in the lateral decubitus position, respectively, on a regular table under general anesthesia. All THAs were performed with cementless stems and cups. All patients undergoing both DAA and Hardinge approaches could leave their beds and were permitted full weight-bearing after drain removal. Patients who underwent THAs using the Dall approach required restricted weight-bearing for 3 weeks to avoid detaching the greater trochanter. Patient information including sex, age, and preoperative diagnosis was collected. The incidence of VTE was compared between DAA and DLA. The patients who underwent THA using DAA were divided into 2 groups (first half and latter half of the period after introduction of DAA), and sex, age, body mass index (BMI), and surgical time were compared between the 2 groups. Additionally, the incidence of VTE was compared among 3 groups: the first half and latter half of DAA, and DLA. Enhanced computed tomography (CT) or lower extremity ultrasound (US) was performed to investigate for VTE at 2 weeks after THA. The sensitivity and specificity of enhanced CT for VTE were reported to be 89–100% and 94–100%, respectively [4–6]. The sensitivity and specificity of lower extremity US for VTE were reported to be 90.1–94% and 98.5–97.3%, respectively [7].

Manual calf massage was performed to maintain venous circulation and increase the blood flow velocity and muscular pumping of the calf by passive ankle motion after THA for VTE prevention according to the method reported by Funayama et al. and Imai et al. [8, 9].

### 2.1. Statistical Analysis

Comparisons between groups were performed using the Mann–Whitney *U* test. Proportions were analyzed using the chi-squared test. All statistical analyses were performed using Statistical Package for the Biosciences software (SPBS version 9.68) [10]. This study was performed with the approval of our institutional ethics committee. The patients and/or their families were informed that their data would be submitted for publication, and they gave their consent.

## 3. Results

Preoperative diagnoses in the DAA group were osteoarthritis (OA; 29 hips), rapid destructive coxarthropathy (RDC; 5 hips), and idiopathic osteonecrosis of the femoral head (ION; 2 hips). Preoperative diagnoses in the DLA group were OA (74 hips), RDC (1 hips), ION (4 hips), and rheumatoid arthritis (RA; 1 hip). The DAA group had a significantly higher percentage of female patients (*p*=0.009). The incidence of VTE was significantly higher in the DAA group (*p*=0.014). The mean surgical time was significantly longer in the DLA group (*p* < 0.001) (Table 1).

There was no significant difference in the sex, age, and BMI between the first half and latter half groups. Surgical time was significantly longer in the first half group than in the latter half group (*p*=0.046) (Table 2). The incidence of VTE in the first half group was higher than in the latter half group and the DLA group (*p*=0.035 and *p*=0.001, respectively). However, there was no difference in the incidence of VTE between the latter half group and the DLA group (*p*=0.923).

## 4. Discussion

This study compared the incidence of VTE after THA using DAA and using DLA. The incidence of VTE after THA was significantly higher after DAA than after DLA. In particular, the incidence of VTE was higher during the first half of the period following introduction of DAA.

The cause of VTE has been described as Virchow's triad: venous stasis, endothelial insult, and hypercoagulability. The surgical approach was one of the causes of venous stasis and endothelial insult. In both posterior and lateral approaches, the combination of femoral flexion, adduction, and internal rotation required for canal preparation causes obstruction of the femoral vein in both the posterior and lateral approaches [11]. On the other hand, the combination of femoral extension, adduction, and external rotation required for canal preparation in the DAA does not cause femoral vein obstruction [12–14]. Therefore, the position for canal preparation during THA using DAA may not be the cause of VTE. However, in the present study, the prevalence of VTE was higher in the DAA group, especially during the first half of the period after introduction of DAA, than in the DLA group. A previous study reported that the femoral venous cross-sectional area and peak flow were significantly decreased in the acetabulum preparation position with a retractor over the anterior wall during acetabular preparation, compared with preincision during THA using DAA [15]. Several studies showed that, because there were leaning curves for THA using the DAA, surgeons who introduced DAA have an increased risk of complications and worse outcomes [16–18]. Considering that VTE cases were concentrated in the first half of the period after DAA introduction and that the surgical time of the first half group was also significantly longer in the present study, VTE was likely to occur due to the longer time of femoral vein occlusion by the retractors. Therefore, the operator should relax retractors when not actively working on acetabular preparation.

To the best of our knowledge, this is the first report to show the possibility of VTE occurrence after THA using DAA, especially in the early period after introducing the DAA. In particular, the present study is a valuable report that shows shortening the preparation time of the acetabulum with retractors can lead to VTE prevention. Therefore, surgeons should become able to prepare the acetabulum in a shorter time using retractors while gaining experience. However, this study had several limitations. First, we did not investigate the degree of hip dysplasia such as “Crowe classification” which may affect the surgical indication of DAA [19]. Second, femoral venous cross-sectional area could not be measured during the operation with ultrasound. Evaluation of blood flow using ultrasound was a useful method; however, it was difficult from the perspective of preventing infection during surgery. Third, the time using a retractor was not measured. Thus, we have to next evaluate the risk of VTE occurrence due to using a retractor by precisely measuring the retractor time and determining the VTE occurrence rate. Fourth, the interobserver and intraobserver agreements of the diagnosis by enhanced CT or lower extremity US were not investigated.

In conclusion, a larger number of VTEs occurred after THA using DAA, especially in the early period than using the DLA. It may be important to shorten the time of using retractors during THA through DAA. In addition, careful monitoring for VTE after surgery and aggressive anticoagulation therapy may be required in the early stage of introducing the DAA until the surgeon becomes familiar with the procedure.

## Figures and Tables

**Table 1:** Patients' characteristics and VTE incidence in the DAA and DLA groups.

	DAA (*n* = 36)	DLA (*n* = 80)	*p* value
Female, *n* (%)	34 (94.4)	59 (73.8)	0.009
Age (years)	65.1 ± 10.8	62.4 ± 11.2	0.265
Preoperative diagnosis			
OA	29	74	
RDC	5	1	
ION	2	4	
RA	0	1	
Surgical time (minutes)	140.1 ± 18.1	169.2 ± 47.6	0.001
The incidence of VTE, *n* (%)	7 (19.4)	4 (5.0)	0.014

VTE: venous thromboembolism, DAA: direct anterior approach, DLA: direct lateral approach, OA: osteoarthritis, RDC: rapid destructive coxarthropathy, ION: idiopathic osteonecrosis of femoral head, RA: rheumatoid arthritis. Data represent mean ± SD or number of patients.

**Table 2:** Comparison of patients' and surgical characteristics according to the period after introducing DAA.

	First half (*n* = 18)	Latter half (*n* = 18)	*p* value
Female, *n* (%)	16 (88.9)	18 (100)	0.146
Age (years)	65.8 ± 10.8	64.4 ± 11.0	0.635
BMI (kg/m^2^)	24.2 ± 2.7	23.7 ± 4.7	0.527
Surgical time (minutes)	147.7 ± 19.7	133.5 ± 14.9	0.046
Incidence of VTE, *n* (%)	6 (33.3)	1 (5.6)	0.035

DAA: direct anterior approach, BMI: body mass index, VTE: venous thromboembolism. Data represent mean ± SD or number of patients.

## Data Availability

The data used to support the findings of this study are available from the corresponding author upon request.

## References

[1] Hill J., Treasure T. (2010). Reducing the risk of venous thromboembolism in patients admitted to hospital: summary of NICE guidance. *BMJ*.

[2] Mont M. A., Jacobs J. J., Boggio L. N. (2011). AAOS clinical practice guideline: preventing venous thromboembolic disease in patients undergoing elective hip and knee arthroplasty. *American Academy of Orthopaedic Surgeon*.

[3] Galakatos G. R. (2018). Direct anterior total hip arthroplasty. *Mo Medical*.

[4] Byun S. S., Kim J. H., Kim Y. J., Chun Y. S., Park C. H., Kim W.-H. (2008). Evaluation of deep vein thrombosis with multidetector row CT after orthopedic arthroplasty: a prospective study for comparison with Doppler sonography. *Korean Journal of Radiology*.

[5] Cham M. D., Yankelevitz D. F., Shaham D. (2000). Deep venous thrombosis: detection by using indirect CT venography. *Radiology*.

[6] Duwe K. M., Shiau M., Budorick N. E., Austin J. H. M., Berkmen Y. M. (2000). Evaluation of the lower extremity veins in patients with suspected pulmonary embolism. *American Journal of Roentgenology*.

[7] Bhatt M., Braun C., Patel P. (2020). Diagnosis of deep vein thrombosis of the lower extremity: a systematic review and meta-analysis of test accuracy. *Blood Advances*.

[8] Funayama A., Kitsuta Y., Fujie A., Tando T., Kanaji A., Toyama Y. (2013). Transition and present status of the prevention of VTE in total hip arthroplasty with manual hand massage of the lower extremities after operation. *Hip Joint*.

[9] Imai N., Ito T., Suda K., Miyasaka D., Endo N. (2017). Manual calf massage and passive ankle motion reduce the incidence of deep vein thromboembolism after total hip arthroplasty. *Journal of Orthopaedic Science*.

[10] Murata K., Yano E. (2002). *Medical Statistics for Evidence-Based Medicine with SPBS User’s Guide*.

[11] Stamatakis J. D., Kakkar V. V., Sagar S., Lawrence D., Nairn D., Bentley P. G. (1977). Femoral vein thrombosis and total hip replacement. *BMJ*.

[12] Binns M., Pho R. (1990). Femoral vein occlusion during hip arthroplasty. *Clinical Orthopaedics and Related Research*.

[13] Stryker L. S., Gilliland J. M., Odum S. M., Mason J. B. (2015). Femoral vessel blood flow is preserved throughout direct anterior total hip arthroplasty. *The Journal of Arthroplasty*.

[14] Warwick D., Martin A., Glew D., Bannister G. (1994). Measurement of femoral vein blood flow during total hip replacement. Duplex ultrasound imaging with and without the use of a foot pump. *The Journal of Bone and Joint Surgery. British Volume*.

[15] Mednick R. E., Alvi H. M., Morgan C. E., Stover M. D., Manning D. W. (2015). Femoral vein blood flow during a total hip arthroplasty using a modified Heuter approach. *The Journal of Arthroplasty*.

[16] Wayne N., Stoewe R. (2009). Primary total hip arthroplasty: a comparison of the lateral Hardinge approach to an anterior mini-invasive approach. *Orthopedic Reviews (Pavia)*.

[17] Woolson S. T., Pouliot M. A., Huddleston J. I. (2009). Primary total hip arthroplasty using an anterior approach and a fracture table. *The Journal of Arthroplasty*.

[18] den Hartog Y. M., Mathijssen N. M. C., Vehmeijer S. B. W. (2016). The less invasive anterior approach for total HIP arthroplasty: a comparison to other approaches and an evaluation of the learning curve-a systematic review. *HIP International*.

[19] Crowe J. F., Mani V. J., Ranawat C. S. (1979). Total hip replacement in congenital dislocation and dysplasia of the hip. *The Journal of Bone & Joint Surgery*.

